# HBx Mediated Increase of DDX17 Contributes to HBV-Related Hepatocellular Carcinoma Tumorigenesis

**DOI:** 10.3389/fimmu.2022.871558

**Published:** 2022-06-16

**Authors:** Mei-Ling Dong, Xu Wen, Xin He, Ji-Hua Ren, Hai-Bo Yu, Yi-Ping Qin, Zhen Yang, Min-Li Yang, Chong-Yang Zhou, Hui Zhang, Sheng-Tao Cheng, Juan Chen

**Affiliations:** Key Laboratory of Molecular Biology for Infectious Diseases, Chinese Ministry of Education, Chongqing Medical University, Chongqing, China

**Keywords:** DDX17, HBx, HCC, metastasis, ZWINT

## Abstract

HBV is strongly associated with HCC development and DEAD-box RNA helicase 17 (DDX17) is a very important member of the DEAD box family that plays key roles in HCC development by promoting cancer metastasis. However, the important role of DDX17 in the pathogenesis of HBV-related HCC remains unclear. In this study, we investigated the role of DDX17 in the replication of HBV and the development of HBV-associated HCC. Based on data from the GEO database and HBV-infected cells, we found that DDX17 was upregulated by the HBV viral protein X (HBx). Mechanistically, increased DDX17 expression promoted HBV replication and transcription by upregulating ZWINT. Further study showed that DDX17 could promote HBx-mediated HCC metastasis. Finally, the promotive effect of DDX17 on HBV and HBV-related HCC was confirmed *in vivo*. In summary, the results revealed the novel role of DDX17 in the replication of HBV and the metastasis of HBV-associated HCC.

## Introduction

Liver cancer remains one of the most important causes of cancer death worldwide. Recently, approximately 700,000 deaths were attributed to liver cancer worldwide (1). Hepatocellular carcinoma (HCC) is the seventh most common cancer in women, the fifth in men (2), and the third leading cause of cancer-related death (3). The risk factors for HCC include autoimmune hepatitis, alcohol abuse, diabetes mellitus, and chronic hepatitis B virus (HBV) infection (4–9). Although operation and antiviral therapies have made significant progress, the median survival of patients with HCC is limited to approximately 6 to 20 months due to metastasis following diagnosis (10). Therefore, elucidation of the mechanism underlying HBV-related HCC metastasis will improve the prognosis of patients with HCC.

DEAD-box proteins, including 38 members in humans, 25 in yeast, and 9 in bacteria are very important members of the ATP-dependent RNA helicase families (11). They are generally essential components of large multiprotein complexes and play roles in RNA metabolism (12, 13). Recent studies have shown that DEAD-box family members play central roles in a variety of cancers and have different mechanisms (14–17). In particular, DEAD-box RNA helicase 17 (DDX17) has attracted substantial attention in cancer research because of its vital biological functions in tumorigenesis, proliferation, and especially, metastasis. The alteration of DDX17 activity reportedly affects pre-mRNA splicing, which is linked to pancreatic ductal adenocarcinoma metastasis and a malignant phenotype (18). A study on uveal melanoma found differential expression of DDX17 in clear-cell renal cell carcinoma (ccRCC) specimens suggests that DDX17 plays a key role in ccRCC metastasis (19). In addition, our previous study found that DDX17 promotes HCC metastasis by regulating the alternative splicing of PXN-AS1 (20). Moreover, an increasing number of studies suggest that the DEAD-box family also plays a role in infection with viruses, including rift valley fever virus (RVFV), dengue virus (DENV), influenza A virus (IVA), and human immunodeficiency virus type 1 (HIV-1) (21–24). Considering that HBV infection, and especially the HBV viral protein X (HBx), is closely related to HCC metastasis, DDX17 may participate in HBx-mediated HCC metastasis.

The viral protein HBx is a multifunctional regulator encoded by HBV that is essential for virus replication. As a key protein regulating HBV replication, HBx has also drawn substantial attention regarding its role in the development and progression of liver cancer. Many studies have shown that HBx can regulate hepatocyte proliferation, regeneration, and apoptosis to promote the progression of HCC (25–28). Debora Salerno and his colleagues showed that in infected hepatocytes, HBx can bind the promoter region of DLEU2 to enhance DLEU2 transcription, induce the accumulation of DLEU2 RNA species and promote the development of HCC (29). Moreover, another study reported that HBx enhances autophagy in HepG2 cells through the PI3K-Akt-mTOR pathway to regulate HCC and potentially promotes cancer metastasis (30). In addition, studies on HCC have suggested that HBx can regulate cancer stem cells to stimulate the proliferation and metastasis of HCC (31). Briefly, HBx functions as an oncogene in the occurrence, development, and metastasis of HCC and we, therefore, wanted to investigate whether DDX17 functions as a bridge between HBV with HCC and regulates HBV-related HCC together with HBx.

In this study, we investigated the role of DDX17 in HBV replication and discovered that DDX17 significantly promoted HBV replication by elevating the expression of ZWINT. Furthermore, we found that DDX17 affected the viral protein HBx to thereby regulate HBV-related HCC metastasis, indicating its great potential as a therapeutic target for HBV and HCC.Materials and Methods

### Cell Culture

HepAD38 cells were obtained from Prof. Ningshao Xia at Xiamen University. Huh-7 and PLC/PRF/5 cells were obtained from the Health Science Research Resource Bank. The HepG2-NTCP cell line was constructed by our laboratory. HepAD38 cells were cultivated in 90% Dulbecco’s modified Eagle medium (DMEM) (Corning, USA) containing 10% fetal bovine serum (FBS) supplemented with 400 μg/mL G418 (Merck, Germany). Huh-7 and PLC/PRF/5 cells were cultured in DMEM supplemented with 10% FBS. HepG2-NTCP cells were cultured in DMEM supplemented with 10% FBS and 2 μg/mL doxycycline. All cell lines mentioned were cultured in a humidified incubator at 37°C and 5% CO2.

## Plasmids, Antibodies and Reagents

The plasmid expressing DDX17 was obtained from Addgene (#19876). The ZWINT expression plasmid was successfully constructed by inserting full-length ZWINT into pcDNA3.1. Plasmids expressing different HBV viral proteins (HBc, preCore/core protein; HBs, S protein; HBx, X protein; and HBp, Pol protein) were constructed by inserting the full-length HBc, HBs, HBx and Pol sequences into pcDNA3.1, which has a Flag tag at the C-terminus. Plasmid pCH9/3091, containing a 1.1-mer HBV genome, was obtained from Prof. Lin Lan (The Army Medical University, Chongqing, China). An HBx-deleted HBV plasmid (HBV-ΔHBx) was obtained by introducing a stop codon at the beginning of the HBx gene based on pCH9/3091, thought to be wild-type HBV (HBV WT).

Short hairpin RNAs targeting DDX17 (shDDX17-1 and shDDX17-2) and a nontargeting shRNA (shCont) were obtained from Shanghai Genechem Company Limited (Shanghai, China), and the target shRNA sequences are provided in **Supplementary Table 1**. Short interfering RNAs (siRNAs) targeting ZWINT (siZWINT-1 and siZWINT-2) and a nontargeting siRNA (siCont) were obtained from Tsingke Biotechnology Company Limited (Beijing, China).

Rabbit anti-DDX17 (19910-1-AP), rabbit anti-ZWINT (12282-2-AP), rabbit anti-THBS1 (18304-1-AP) and rabbit anti-KIF23 (28587-1-AP) antibodies were obtained from Proteintech (Chicago. USA). The rabbit anti-Flag (#14793S) antibody was purchased from Cell Signaling Technology (Boston, USA), and the mouse anti-HBc was kindly provided by Dr. XueFei Cai (Chongqing Medical University, China). The rabbit anti-SLA2 (27794-1) antibody was purchased from Signalway Antibody (California, USA), and the mouse anti-GAPDH (MB001) antibody was purchased from Bioworld Technology (USA).

Doxorubicin was purchased from Solarbio (ID0670, Beijing, China), and G418 was purchased from BioFroxx (1150GR001, Germany).

### Quantitative Reverse Transcription PCR (qRT–PCR)

Total RNA was extracted from cells and tissues using TRNzol (DP424, TIANGEN, Beijing, China) and reverse transcribed into cDNA using a Prime Script qRT–PCR kit (Bio–Rad, KR116-02, Bio–Rad, California, USA). The cDNA was amplified by qRT–PCR with Fast Start Universal SYBR Green Master and the relative levels of viral RNA and mRNA were determined by the 2^-△△CT^ method; beta-actin served as the internal control. The primer sequences used in this study are shown in **Supplementary Table 2**.

### HBV DNA Extraction and Quantitative PCR

Equal numbers of cells were lysed with 500 μL of lysis buffer at 37°C for 30 min. After centrifugation, the supernatant was added to a new 1.5 ml tube and incubated with 4 μL of DNase I and 5 μL of 1 M MgCl_2_ at 37°C for 4 hours. After precipitation with 35% PEG8000, proteinase K was added to the tube and incubated with HBV DNA at 45°C overnight. Then, HBV DNA was extracted with phenol chloroform, washed with 70% ethanol, and dissolved in H_2_O. Absolute quantification of the extracted HBV DNA was performed by using Fast Start Universal SYBR Green Master Mix (06924204001, Roche, Mannheim, Germany). The primer sequences are listed in **Supplementary Table 2**.

### Southern Blot

HBV DNA was separated on 0.9% agarose gels and transferred onto nylon membranes (11417240001, Roche, Mannheim, Germany). After fixation by UV crosslinking and prehybridization, the membranes containing HBV DNA were hybridized with a digoxigenin-labelled HBV-specific probe overnight at 42°C and then washed with different concentrations of SSC containing 0.1% SDS. The membranes were incubated in a blocking solution at 37°C for 30 min and then incubated with an anti-Dig antibody at 37°C for 30 min. The signal was observed by exposure to an X-ray film.

### Northern Blot

To analyze the total RNA extracted by TRNzol reagent, the DIG Northern Starter Kit (12039672910, Roche, Mannheim, Germany) was used according to the manufacturer’s protocol. Briefly, the total RNA extracted from cells was separated by 1.4% formaldehyde-agarose gel electrophoresis and washed with 20×SSC for 15 min at room temperature. Then, the RNA samples were transferred onto nylon membranes and fixed by UV crosslinking. The membranes were hybridized with a DIG-labelled HBV RNA probe overnight at 68°C and then washed with different concentrations of SSC containing 0.1% SDS. After incubation in blocking solution at 37°C for 30 min, the membranes were incubated with an anti-digoxin antibody at 37°C for 30 min. The signal was detected by using X-ray film, and 28S and 18S RNA served as internal controls.

### ELISA

The secretion level of HBV surface antigen (HBsAg) in the cell culture medium was measured by a commercial ELISA kit (KHB, China) according to the manufacturer’s protocol. The cell culture medium was collected and centrifuged at 2,000 g for 3 min to remove the cell debris. Then, 75 μL of each sample was subjected to ELISA, and the absorbance at 450 nm was measured. Negative and positive controls were included with samples.

### Western Blot

Cells were collected and subjected to total protein extraction by using RIPA lysis buffer containing protease inhibitors (04693159001, Roche, Mannheim, Germany). The protein concentration was determined by the BCA protein assay (1859078, Roche, Mannheim, Germany), and 30 μg of protein was denatured for 10 min at 95°C.

After separation by SDS–PAGE, the proteins were transferred onto polyvinylidene fluoride membranes (IPVH00010, GE Healthcare, Buckinghamshire, UK). After blocking with 5% nonfat milk for 2 hours, the membranes were incubated with relevant primary antibodies (anti-DDX17 protein 1:2000; anti-ZWINT protein 1:1000; anti-Flag 1:3000, anti-HBc 1:1000, anti-THBS1 1:3000, anti-KIF23 1:3000, anti-SLA2 1:3000, anti-GAPDH 1:5000) overnight at 4°C and then with the appropriate secondary antibody. The blots were visualized by ECL Western blot reagents (WBKLS0500, Millipore. Massachusetts, USA), and GAPDH served as an internal control.

### Coimmunoprecipitation

Cells were lysed with RIPA lysis (containing protease inhibitor) at 4°C for 20 min. After centrifugation at 16,000 g for 5 min at 4°C, the protein concentration was determined by the BCA assay (1859078, Roche, Mannheim, Germany). Lysates containing 600 μg of total protein were precleared with protein A/G magnetic beads (LSKMAGG10, Merck Millipore, Darmstadt, Germany) for 1 hour at 4°C. Next, the protein complex was incubated with the relevant primary antibody overnight at 4°C. The next day, after washing with PBS/0.1% Triton-100 three times, the protein A/G magnetic beads were added to the complex lysates and rotated for 2 hours at room temperature. Finally, the protein-antibody-bead complexes were eluted with 3× SDS loading buffer and subjected to Western blotting.

### Virus Collection and Infection

HBV virion particles were obtained from the medium of HepAD38 cells. Briefly, the supernatants of HepAD38 cells were collected and then mixed with 4% PEG8000. The samples were then rotated gently at 4°C and dissolved overnight. The complexes were centrifuged at 4,000 rpm for 30 min at 4°C to collect HBV particles. The pellet was resuspended in Opti-MEM (Gibco) at a 100-fold concentration, and the viral titre was quantified by measuring HBV DNA by qPCR. For infection, cells in collagen-coated wells were infected with HBV particles diluted in William’s medium supplemented with 10% FBS, 2 mM sodium pyruvate, 200 μM L-glutamine, 2.5% dimethyl sulfoxide (DMSO), 4% PEG8000 and 1% P/S. HepG2-NTCP cells were infected with a total of 1,000 genome equivalents/cell of HBV particles in 4% PEG8000 for 24 hours and then washed twice with PBS before use in further experiments.

### *In Vitro* Assays of Transwell Migration

Transwell chambers (24-well plate, 8 m pores; cat# 356234, BD Biosciences) were used to assess the migration abilities of cells. Briefly, 2×10^5^ HCC cells were resuspended in DMEM and seeded into the upper chamber and medium containing 10% FBS was added to the lower chamber. The cells were further cultured at 37°C/5% CO2. Then, the migrated cells were fixed with 95% methanol and the cells in the upper chamber were scraped with a cotton swab, while those in the lower chamber were stained with 0.1% crystal violet dye. The number of migrated cells was counted under an inverted microscope.

### Immunohistochemistry

The liver tissues of mice were fixed with formalin and embedded in paraffin. After deparaffinization in xylene, the sectioned tissues were microwaved and heated in sodium citrate buffer (10 mmol/L, pH 6.0). Then, the specimens were incubated with the indicated primary antibodies at 4°C overnight. Following counterstaining with the appropriated secondary antibody for 30 min, diaminobenzidine (DAB) staining was used to detect tissue immunoreactivity (Dako, Carpinteria, CA). Haematoxylin was used for counterstaining.

### Construction of a Mouse Model of HBV Infection Using HBV Recombinant (r) cccDNA

C57BL/6 mice were purchased from the Laboratory Animal Center of Chongqing Medical University (Chongqing, China) and fed under pathogen-free conditions. Four micrograms of prcccDNA and four micrograms of Cre were diluted to 8% of the mouse body weight with PBS and then hydrodynamically injected into the mice *via* their tail vein within 5 to 8 s. After one week, the mice were randomly assigned to either the negative group (shCont, 2E+11 v.g./mL, n=8) or the treatment group (shDDX17, 2E+11 v.g./mL, n=8) and sacrificed at the indicated time points. Serum was collected every four days and stored at -80°C for further study. Liver tissues were collected for intrahepatic HBV RNA and HBV DNA detection. All animal studies conformed to the Animal Research: Reporting of *In Vivo* Experiments (ARRIVE) guidelines and were approved by the Laboratory Animal Center of Chongqing Medical University.

### Clinical Samples

With informed consent, human HCC liver tissues were collected from 24 patients at The First Affiliated Hospital of Chongqing Medical University. All patients who provided liver tissues were diagnosed with HBV-related HCC and were HBsAg-positive. Metastasis was observed in 12 of the 24 patients. All tissues were frozen immediately upon resection and stored in liquid nitrogen for further investigation. This study was approved by The Clinical Research Ethics Committee of Chongqing Medical University.

### Statistical Analysis

The results are expressed as the mean ± SD from at least three independent experiments. Statistical analyses were performed using either the Mann–Whitney U test or one-way ANOVA. P < 0. 05 was considered significant (*P < 0. 05, **P < 0. 01). All statistical analyses were conducted using GraphPad Prism 8 (GraphPad) software (GraphPad Software, Inc., La Jolla, CA).

## Results

### HBV Promotes DDX17 Expression and May Be Involved in HCC Metastasis

**Figure 1:**
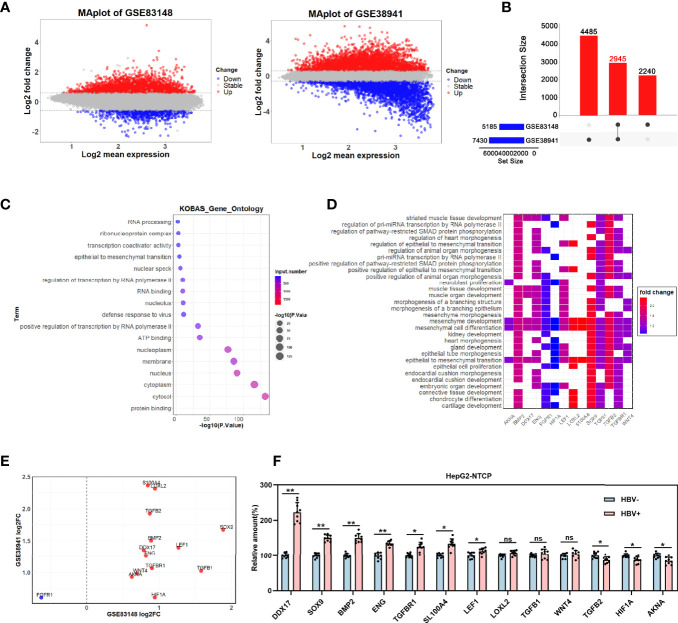
The effect of HBV on DDX17 expression were analyzed in GEO database. **(A–E)** Dataset GSE83148 and GSE38941 were download from GEO database and subjected to bioinformatics analysis. **(A)** Volcano plots show the potential differentially expressed genes in GSE83148 and GSE38941(log2FC>0.585, adjusted p < 0.05). **(B)** The Venn diagrams reveal the differentially expressed genes and the co-expressed genes among the 2 cohorts (fold change value >2). **(C)** Functional enrichment analysis shows the co-up-regulated and co-down-regulated genes. **(D)** Signaling pathway analysis for 14 candidate genes. **(E)** Co-expression of 14 genes in GSE83148 and GSE838941. **(F)** Real-time PCR analysis for the 13 genes expression levels in HBV-infected HepG2-NTCP cells. β-actin was used as an internal quantitative control. ns means no significance, *P < 0.05, **P < 0.01.

To explore the molecular mechanisms underlying HBV-related HCC metastasis, we analyzed the differentially expressed genes (DEGs) in the GSE83148 (comprising 122 HBV-infected liver tissues and 6 normal liver tissues) and GSE38941 (comprising 17 HBV-associated acute liver failure tissues and 10 normal liver tissues) GEO datasets. The 2 datasets were available on the Gene-Cloud of Biotechnology Information (GCBI) bioinformatics analysis platform and the DEGs were identified by using the GEO2R online analysis tool (log2FC>0.585, adjusted p <0.05). GSE83148 contained a total of 5,185 DEGs, including 4,646 upregulated and 539 downregulated genes, whereas GSE38941 contained 7,430 DEGs, including 4,224 upregulated and 3,206 downregulated genes (**Figure 1A**). As shown in **Figure 1B**, 2,945 DEGs (fold change value >2) were co-expressed in these two datasets (**Figure 1B**). To further analyze the functional roles of the co-expressed DEGs in the progression of HBV infection, the 2,945 genes were subjected to pathway analysis. Notably, 14 genes were enriched in epithelial to mesenchymal transition-related pathways (**Figures 1C, D**), indicating their potential involvement in HBV-mediated tumor metastasis. The expression levels of the 14 co-expressed genes (excluding FGFR1) were upregulated in both GSE83148 and GSE838941 (**Figure 1E**). Then, the effect of HBV infection on the expression levels of these 13 upregulated genes was further investigated in HBV-infected HepG2-NTCP cells, revealing that DDX17 expression was obviously increased (**Figure 1F**).

**Figure 2:**
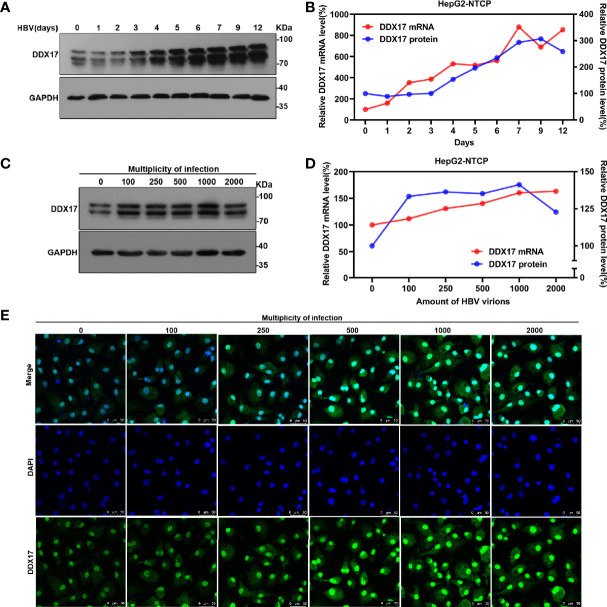
The effect of HBV on DDX17 expression were analyzed in HBV-infected HepG2-NTCP cells. **(A, B)** HepG2-NTCP cells were infected with HBV (1, 000 genome equivalents/cell) and harvested at indicated days. Real time PCR and western blot measured the mRNA and protein level of DDX17, respectively. **(C, D)** HepG2-NTCP cells were infected with series dose of HBV particle and harvested at 5 days post-infection. Real time PCR and western blot measured the mRNA and protein level of DDX17, respectively. **(E)** Endogenous DDX17 level in HepG2-NTCP cells which infected with series doses of HBV particles was determined by immunofluorescence staining.

To broadly investigate the association between HBV infection and DDX17, the effect of HBV infection on DDX17 expression was first analyzed in HBV-infected HepG2-NTCP cells. Based on real-time PCR and Western blot analyses, both the mRNA and protein levels of DDX17 were upregulated in HBV-infected HepG2-NTCP cells on the indicated day after infection (**Figures 2A, B**). In addition, HepG2-NTCP cells were infected with increasing HBV particles at increasing MOIs (100, 250, 500, 1000 and 2000 virion genome equivalents/cell), and both the mRNA and protein levels of DDX17 were examined. As expected, the HBV-induced increase in DDX17 expression was dependent on the dose of the HBV particles (**Figures 2C, D**). Similar results were observed in HepG2-NTCP cells infected with HBV at a series of MOIs by immunofluorescence analysis (**Figure 2E**). Collectively, these data suggested that HBV upregulated the expression of DDX17.

### The Viral Protein HBx Is Responsible for DDX17 Upregulation

**Figure 3:**
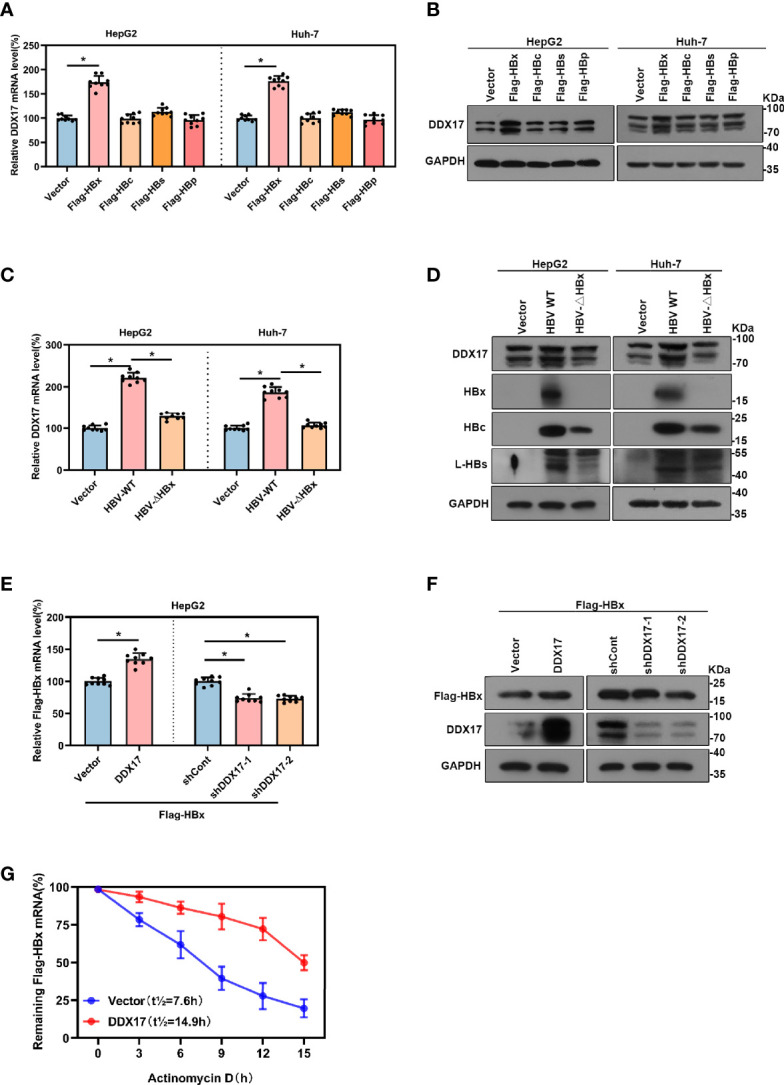
There was a positive correlation between Viral protein HBx and DDX17. **(A, B)** The vector, Flag-HBx, Flag-HBc, Flag-HBs or Flag-HBp plasmids were transfected into HepG2 cells and Huh-7 cells. Real-time PCR and Western blot examined the mRNA and protein levels of DDX17. β-actin and GAPDH were used as the internal quantitative controls respectively. **(C, D)** The vector, HBV WT or HBV-ΔHBx plasmids were transfected into HepG2 and Huh-7 cells. Real-time PCR examined the mRNA evels of DDX17 and Western blot examined the protein levels of DDX17, HBx, HBc and HBs. β-actin and GAPDH were used as the internal quantitative controls, respectively. **(E, F)** The vector or DDX17 plasmids were co-transfected with Flag-HBx into the HepG2 cells; the short hairpin RNAs targeting DDX17 (shDDX17-1 and shDDX17-2) or nontargeting shRNA (shCont) were co-transfected with Flag-HBx into the HepG2 cells. Real-time PCR and Western blot examined the mRNA and protein levels of Flag-HBx. **(G)** DDX17 overexpression in HepG2 cells followed by treatment with 5ug/mL actinomycin D at the indicated times. β-actin and GAPDH were used as the internal quantitative controls, respectively. *P < 0.05.

Although our data showed that DDX17 expression was upregulated by HBV, the underlying mechanism is not known. To further investigate this, the effects of viral proteins (HBx, HBc, HBs, HBp) on DDX17 expression were first determined. Plasmids expressing the different HBV viral proteins were transfected into HepG2 or Huh-7 cells, and the DDX17 expression levels were examined by real-time PCR and Western blot. Fortunately, DDX17 expression was obviously increased in cells expressing HBx but not in those expressing the other HBV proteins (**Figures 3A, B**). To further elucidate whether HBx mediates the impact of HBV on DDX17 expression, we generated an HBx-deficient virus (HBV-ΔHBx) in which the expression of the X gene was abrogated. Decreased levels of DDX17 were observed in cells infected with HBV-ΔHBx compared to those infected with HBV WT. The infection with HBx-deficient virus only reduced the level of HBx but did not affect the expression of other viral proteins (**Figures 3C, D**). These data revealed that the expression of DDX17 was enhanced by HBx. We further studied the functional role of DDX17 in HBx expression. As expected, DDX17 overexpression upregulated the expression of HBx, while DDX17 depletion downregulated HBx expression (**Figures 3E, F**). Concordantly, the expression of other viral proteins is not affected after DDX17 overexpression or deletion (**Figures S1A–C**). To investigate the potential mechanism underlying DDX17 regulated the expression of HBx, we co-transfected the DDX17 overexpression plasmid with Flag-HBx into HepG2 cells and it treated with actinomycin D to block transcription and measured decay of existing mRNAs by performing time-course real-time PCR. The results revealed that DDX17 could enhance HBx expression by stabilizing the half-life of HBx mRNA, indicating that DDX17 could promote the HBx post-transcriptional by regulating HBx mRNA stability (**Figure 3G**). Altogether, these results suggests that DDX17 and HBx positively interact.

### Increased DDX17 Expression Induced by HBx Promotes HBV Transcription and Replication

**Figure 4:**
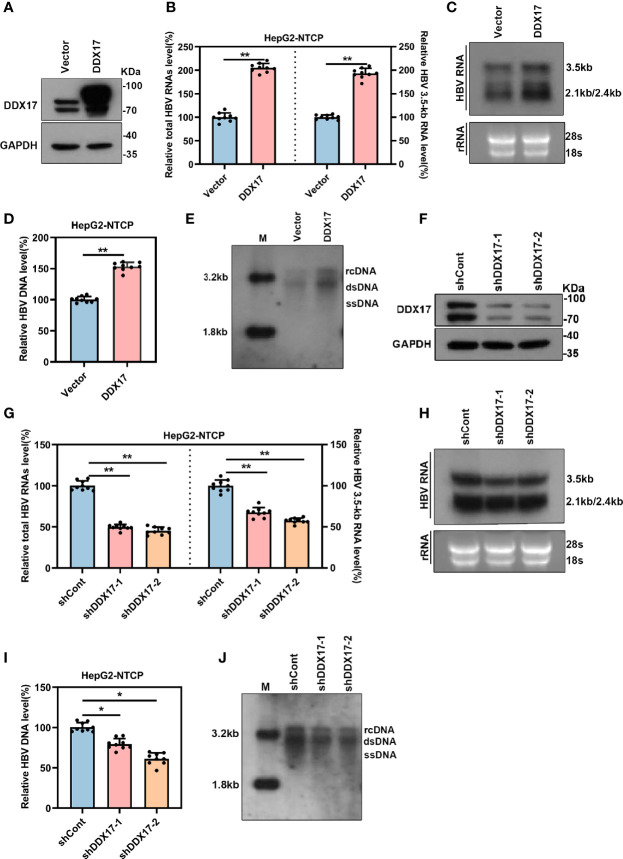
The expression of DDX17 regulates HBV transcription and replication. The vector or DDX17 plasmids and lentivirus expressing shRNA targeting DDX17 or shCont transfected into HepG2-NTCP cells that were infected with 1,000 genome equivalents/cell of HBV particles in the presence of 4% PEG8000 for 24 h. Total HBV RNAs, 3.5-kb RNA, and HBV DNA were analyzed at day 5 after plasmid transduction. **(A)** The expression of DDX17 after transfected with plasmid expressing DDX17 and empty vector in HBV-infected HepG2-NTCP cells were access by using Western blot assay. GAPDH was used as the internal quantitative control. **(B)** Real-time PCR was used to analyze the levels of total HBV RNAs and 3.5-kb RNA. β-actin was used as the internal quantitative control. **(C)** Northern blot assay hybridization of HBV RNAs by using DIG-labeled plus- or minus-strand-specific HBV riboprobe with a length of 1000 bp. Ribosomal RNAs (28S and 18S) were used as loading control. **(D, E)** Real-time PCR and Southern blot assay examined the levels of HBV DNA replicative intermediates. **(F)** The expression of DDX17 after transfected with negative control (shCont) or shRNA (shDDX17-1 and shDDX17-2) in HBV-infected HepG2-NTCP cells were accessed using Western blot assay. GAPDH was used as the internal quantitative control. **(G, H)** Real-time PCR and Northern blot assay were used to analyze the effect of DDX17 knockdown on HBV RNAs in HBV-infected HepG2-NTCP cells. **(I, J)** Real-time PCR and Southern blot assay were used to analysis the effect of DDX17 knockdown on HBV DNA replicative intermediates in HBV-infected HepG2-NTCP cells. *P < 0.05, **P < 0.01.

The above data revealed that DDX17 expression was upregulated by HBx. However, the effect of increased DDX17 expression induced by HBx on HBV was still unclear. To determine whether HBV RNA transcription and DNA replication was also regulated by DDX17, HepG2-NTCP cells were infected with HBV particles before transduction with a plasmid expressing DDX17, and the efficiency was validated by Western blot (**Figure 4A**). Real-time PCR revealed that the levels of total HBV RNA and 3. 5-kb RNA was significantly increased by DDX17 overexpression (**Figure 4B**). Northern blot analysis also confirmed increased levels of HBV 3. 5-, 2. 4- and 2. 1-kb RNA after DDX17 overexpression (**Figure 4C**). Furthermore, the levels of HBV DNA replicative intermediates were determined by real-time PCR and Southern blot, both of which revealed that ectopic DDX17 expression markedly increased the levels of HBV DNA replicative intermediates (**Figures 4D, E**). Taken together, these results demonstrated that overexpression of DDX17 enhanced HBV transcription and replication.

To systematically confirm whether endogenous DDX17 exerts a similar ability in HBV-infected cells, HepG2-NTCP cells were infected with HBV particles before transduction with lentiviruses expressing short hairpin RNAs (shRNAs) targeting DDX17 (shDDX17-1 and shDDX17-2), and the transduction efficacies were validated by Western blot (**Figure 4F**). In contrast, real-time PCR and Northern blot analysis revealed that the levels of total HBV RNA and 3. 5-kb RNA were significantly downregulated in HBV-infected HepG2-NTCP cells expressing shDDX17 (**Figures 4G, H**). DDX17 suppression also resulted in decreased levels of HBV DNA replicative intermediates (**Figures 4I, J**). Overall, these results indicate that DDX17 plays a vital role in promoting HBV transcription and replication.

### DDX17 Promotes HBV Transcription and Replication by Upregulating ZWINT

**Figure 5:**
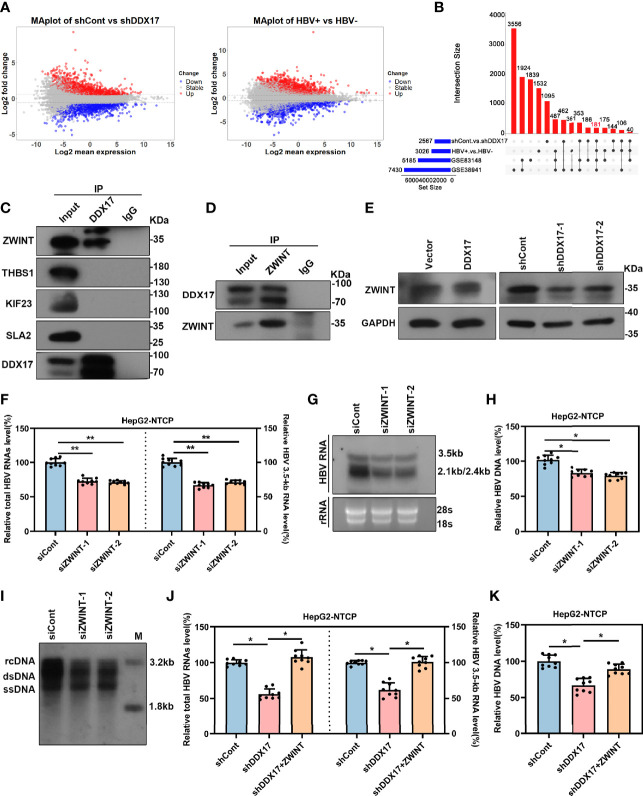
DDX17 promotes HBV transcription and replication by upregulating ZWINT. **(A)** Volcano plot for potential DEGs in two RNA-seq datasets (HBV-infected HepG2-NTCP cells with or without DDX17 knockout; liver tissue from HCC patients with or without HBV infection) (log2FC>0.585, p value<0.05). **(B)** The Venn diagram shows 181 co-expressed genes were identified among GSE83148, GSE38941 and two RNA-seq data. **(C)** The co-immunoprecitation assay (Co-IP) analysis showing the interactions between DDX17 and its candidate interacting proteins. **(D)** Co-IP assay was performed with anti-DDX17 antibody in HBV-infected HepG2-NTCP cells transfected plasmid expressing ZWINT. **(E)** DDX17 regulated ZWINT protein expression in HBV-infected HepG2-NTCP cells measured by western blot assay. GAPDH was used as an internal quantitative control. **(F, G)** Real-time PCR and Northern blot assay were used to analysis the effect of ZWINT knockdown on HBV RNAs in HBV-infected HepG2-NTCP cells. **(H, I)** Real-time PCR and Southern blot assay were used to analysis the effect of ZWINT knockdown on HBV DNA replicative intermediates in HBV-infected HepG2-NTCP cells. **(J, K)** Reintroduction of ZWINT antagonized the effect of DDX17 knockdown on HBV transcription and replication. Total HBV RNAs, 3.5-kb RNA **(J)** and HBV DNA replicative intermediates **(K)** were extracted and examined by real-time PCR. *P < 0.05, **P < 0.01.

To better elucidate the potential molecular mechanism by which DDX17 promotes HBV infection, we assessed the differentially expressed genes regulated by DDX17 in HBV-infected HepG2-NTCP cells with or without DDX17 knockdown by using RNA-seq. A log2 FC of 0.585 and an adjusted p value <0.05 were set as the threshold criteria. A total of 2,567 DEGs were identified, including 1,178 upregulated genes and 1,389 downregulated genes (**Figure 5A**, left panel). The DEGs in liver tissues from HCC patients with and without HBV infection were also analyzed by using RNA-seq, revealing 3,026 DEGs, including 1,745 upregulated genes and 1,281 downregulated genes (**Figure 5A**, right panel). Then, 181 DEGs were obtained by overlapping the two RNA-seq datasets with the two GEO datasets shown in **Figure 1A** (GSE83148 and GSE838941) (**Figure 5B**). Furthermore, the protein interactions between DDX17 and the 181 candidate genes were analyzed by the STRING database, revealing that DDX17 might interact with ZWINT, THBS1, KIF23 and SLA2 (**Figure S2A**). To determine whether DDX17 physically interacts with the four proteins, we performed a reciprocal coimmunoprecipitation (Co-IP) assay, revealing that DDX17 coprecipitated with ZWINT but not with THBS1, KIF23, or SLA2 (**Figures 5C, D**). In addition, we further analyzed the effect of DDX17 on the expression level of ZWINT by real-time PCR and Western blot, demonstrating that silencing DDX17 led to a marked reduction in the ZWINT mRNA and protein levels. In contrast, compared with that in control cells, the expression of ZWINT was markedly increased in cells overexpressing DDX17 (**Figure 5E**; **Figure S2B**).

The above data indicates that the expression of ZWINT is regulated by DDX17, and we next sought to determine whether the expression level of ZWINT affects HBV transcription and replication. To do this, we overexpressed ZWINT in HBV-infected HepG2-NTCP cells and real-time PCR showed that ZWINT overexpression markedly increased the levels of total HBV RNAs and 3. 5-kb RNA. Moreover, Northern blot analysis suggested that ZWINT increased the levels of 3. 5-, 2. 4- and 2. 1-kb HBV RNA (**Figures S2A–C**). In addition, real-time PCR and Southern blotting further demonstrated that ectopic ZWINT expression markedly increased the levels of HBV DNA replicative intermediates (**Figures S3D, E**). ZWINT depletion led to significant decreases in the HBV RNA and DNA levels (**Figures 5F–I**; **Figure S3F**), indicating that silencing ZWINT inhibited HBV transcription and replication.

Next, we further confirmed the involvement of ZWINT in the modulation of DDX17-mediated HBV transcription and replication. We first investigated the effect of ZWINT overexpression on DDX17-silenced cells by examining the levels of total HBV RNA, 3. 5-kb RNA and HBV DNA. As expected, ZWINT overexpression significantly restored the inhibition of HBV transcription and replication in DDX17 knockdown cells (**Figures 5J, K**). In short, we revealed that DDX17 facilitated HBV transcription and replication in a ZWINT-dependent manner.

### DDX17 Promotes HBV Transcription and Replication *In Vivo*

**Figure 6:**
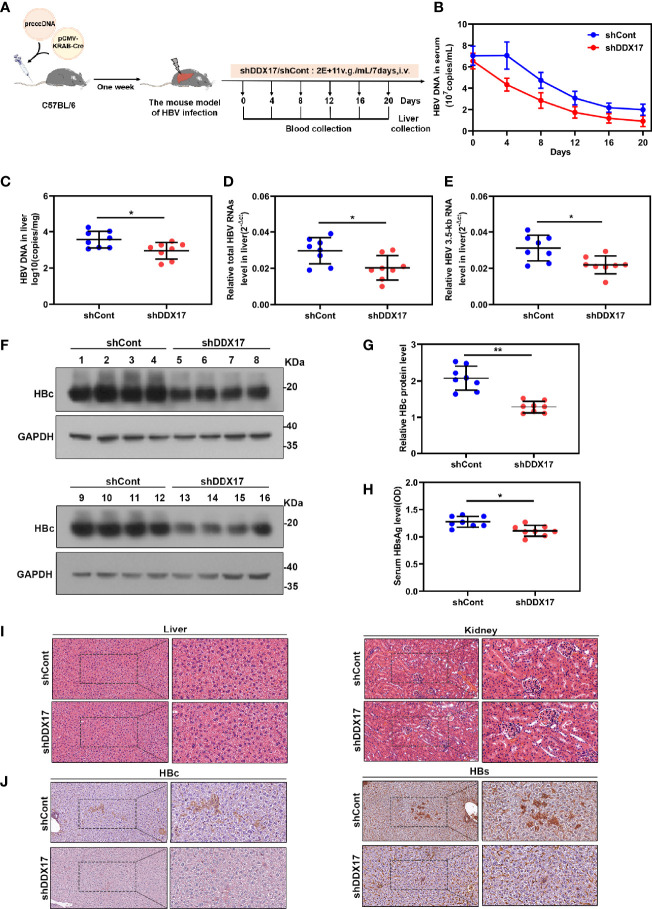
DDX17 promotes HBV transcription and replication *in vivo*. **(A)** Flow chart explaining the way and concentration of AAV-shDDX17 administration as well as the intervals of vein blood collection. The mouse model established by injecting prcccDNA and pCMV-KRAB-Cre from tail vein. The mouse model of HBV infection involving HBV recombinant (r) cccDNA constructed successfully were randomly assigned to 2 groups (n = 8 per group). **(B)** Real-time PCR was subjected to detect the serum level of HBV DNA. **(C)** Real-time PCR was subjected to detect the level of HBV DNA in liver tissue. **(D, E)** Real-time PCR was subjected to detect the total HBV RNAs and 3.5-kb RNA levels in liver tissue, β-actin was used as the internal quantitative control. **(F, G)** The levels of HBc protein were measured by Western blot assay. **(H)** ELISA assay was subjected to determine the level of HBsAg in serum during the treatment. **(I)** Abnormal pathological changes in the two groups were determined by HE staining. **(J)** The expression of HBs and HBc in liver tissues were analyzed by immunohistochemistry. *P < 0.05, **P < 0.01.

Although the inhibitory effect of DDX17 depletion on HBV infection cell models has been demonstrated, its functional implications *in vivo* are still unknown. To evaluate the functional roles of DDX17 *in vivo*, we constructed a mouse model of HBV infection utilizing HBV (r) cccDNA using site-specific DNA recombination. Mice from each group were treated with 2E+11v. g./mL AAV-shDDX17 or AAV-shCont and then sacrificed on the indicated day post injection for a series of analyses (**Figure 6A**). Consistent with the *in vitro* observations, DDX17 depletion markedly inhibited HBV infection. First, the transduction efficiency of the AAV-based viral vector in the mice model *in vivo* was confirmed by Western blot (**Figure S4A**). Meanwhile, DDX17 depletion markedly reduced the serum levels of HBV DNA during treatment, as determined by real-time PCR (**Figure 6B**). The animals were sacrificed after 20 days of treatment, and liver tissues were collected to evaluate the HBV RNA and HBV DNA levels in the mouse liver. Consistently, real-time PCR confirmed that the levels of intrahepatic HBV DNA, total HBV RNAs and 3. 5-kb RNA were significantly reduced after AAV-shDDX17 treatment (**Figures 6C–E**). In addition, Western blot assays indicated that DDX17 depletion reduced the protein expression of HBc (**Figures 6F, G**). Compared with those in control mice, DDX17-depleted mice had significantly decreased serum levels of HBsAg as determined by ELISA (**Figure 6H**). Moreover, no abnormal pathological changes were observed by HE staining in either group (**Figure 6I**). Next, the expression levels of HBsAg and HBcAg in liver tissue were measured by immunohistochemistry, demonstrating that DDX17 depletion apparently reduced their expression in liver tissue (**Figure 6J**). Taken together, these results strongly confirmed that DDX17 depletion repressed HBV infection *in vivo*.

### Increased Expression of DDX17 Mediated by HBx Promotes the Metastasis of HBV-Related HCC

**Figure 7:**
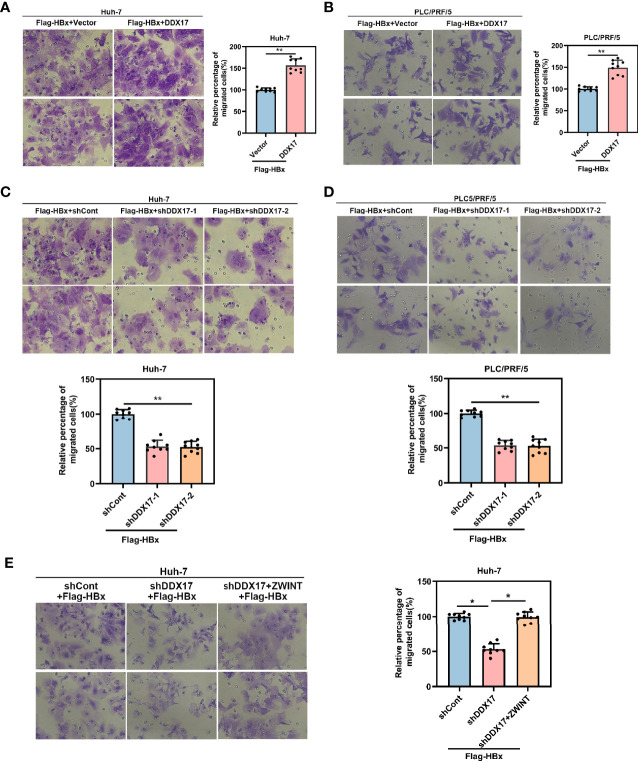
DDX17 could facilitate HBx-mediated HCC migration. **(A, B)** Migratory ability was assessed by transwell assays in Huh-7 and PLC/PRF/5 cells with DDX17 overexpression. **(C, D)** Migratory ability was assessed by transwell assays in Huh-7 and PLC/PRF/5 cells with DDX17 knockout. **(E)** The effects of decreased DDX17 meanwhile upregulation of ZWINT in Huh-7 cells for migration analyzed by transwell assays. *P < 0.05, **P < 0.01.

To further investigate the functional role of DDX17 in the development of HBV-related HCC mediated by HBx, we co-transfected the DDX17 overexpression plasmid with Flag-HBx into HCC cells (Huh-7 and PLC/PRF/5). Increased cell migration was observed in both cell lines, which indicated that DDX17 facilitated HBx-mediated HCC migration (**Figures 7A, B**). To better understand the association between the HBx-mediated increase in DDX17 expression and HCC cell migration, shRNAs targeting DDX17 (shDDX17-1 and shDDX17-2) were co-transfected with Flag-HBx into Huh-7 and PLC/PRF/5 cells. Transwell assays confirmed that in contrast to DDX17 overexpression, the effect of HBx on the migration of Huh-7 and PLC/PRF/5 cells was abolished by DDX17 depletion (**Figures 7C, D**). Moreover, as DDX17 facilitated HBV transcription and replication in a ZWINT-dependent manner, we co-transfected the shRNAs targeting DDX17 (shDDX17) and ZWINT overexpression plasmid with Flag-HBx into Huh-7 cells to assess the involvement of ZWINT in DDX17-mediated HBV in relation to HCC migration. As expected, the transwell assay confirmed that the inhibition of cell migration induced by the absence of DDX17 was rescued by ZWINT overexpression (**Figure 7E**). Meanwhile, we co-transfected the DDX17 overexpression plasmid and siRNAs targeting ZWINT (siZWINT) with Flag-HBx into Huh-7 cells and the results show that knockdown of ZWINT resulted in the disappearance of DDX17 promoting effect on cell migration (**Figure S5A**). In short, we demonstrated that DDX17 facilitated HBx-mediated HCC migration.

### DDX17 Expression Is Associated With HBV-Related HCC Metastasis in Clinical Samples

**Figure 8:**
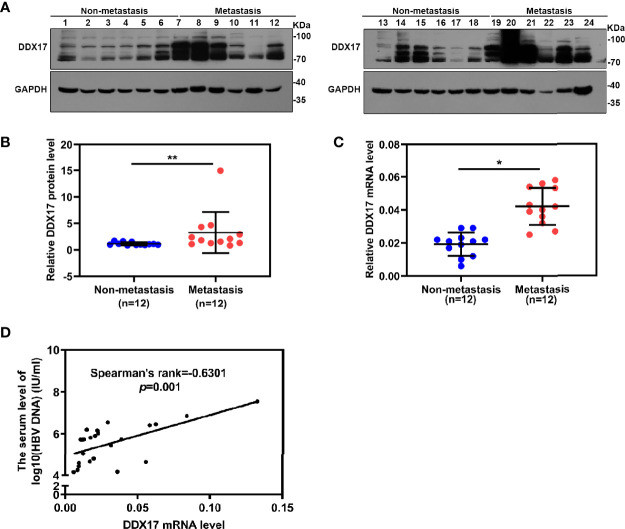
DDX17 play an important role in HBV-related HCC metastasis. **(A, B)** Western blot assay was subjected to examined the protein level of DDX17 in HBV-related HCC tissue samples, GAPDH was used as internal quantitative controls. **(C)** Real-time PCR was subjected to examined the mRNA level of DDX17 in HBV-related HCC tissue samples, β-actin was used as the internal quantitative controls. **(D)** Correlation between the serum HBV DNA levels and the liver DDX17 levels in HBV-related HCC patients. HBV DNA levels was log10-transformed. Correlation coefficients (r) and two-tailed P values were evaluated by Pearson’s test. *P < 0.05, **P < 0.01.

To further confirm the clinical significance of DDX17 expression in HBV-related HCC, we recruited 24 patients diagnosed with HBV-related HCC in this study and divided them into two groups based on their metastatic status (metastatic and nonmetastatic groups). The clinical and virological characteristics of these patients are summarized in **Table 1**. To investigate the potential role of DDX17 in HBV-related HCC, we detected the DDX17 protein levels by Western blotting, revealing increased protein expression levels of DDX17 in the metastatic group compared to the nonmetastatic group (**Figures 8A, B**). Concordantly, real-time PCR revealed that compared to the nonmetastatic group, the mRNA level of DDX17 in the metastatic group was upregulated significantly (**Figure 8C**), which suggests that DDX17 plays a positive role in HBV-mediated HCC metastasis. Moreover, the correlationship between DDX17 and HBV DNA was analyzed and the data showed a significantly positive correlation between the liver DDX17 mRNA levels and serum HBV DNA levels in patients diagnosed with HBV-related HCC (Pearson correlation coefficient r = 0.6301, *P* =0.001; **Figure 8D**). Collectively, the above results support that DDX17 plays an important role in HBV-mediated HCC metastasis.

**Table 1:** Clinical and virological characteristics of the subjects enrolled in the study.

Patient	Age (y)	Gender (F/M)	HBV DNA (IU/ml)	HBsAg (IU/ml)	ALT (IU/ml)	Metastasis (Yes/No)	Differentiation grade
1	76	F	2.56E+06	1772	64	Y	G1
2	60	M	7.03E+06	2350	23	Y	G2
3	46	M	3.58E+07	2254.55	29	Y	G4
4	24	M	2.87E+05	37.52	50	Y	G2
5	43	M	5.50E+05	1214	34	Y	G2
6	52	F	5.51E+05	834.34	62	Y	G2
7	71	F	1.51E+04	2040	20	Y	G2
8	50	M	2.87E+06	1913.91	45	Y	G2
9	42	M	4.62E+04	2486	41	Y	G1
10	62	F	1.57E+06	105	33	Y	G1
11	42	M	4.37E+04	5740	23	Y	G3
12	61	M	6.26E+04	2090	37	Y	G2
13	46	M	7.89E+05	20.33	33	N	G1
14	53	M	1.46E+06	1587	83	N	G1
15	39	M	6.60E+05	3101.42	171	N	G4
16	58	M	2.78E+04	1734	111	N	G2
17	40	M	3.52E+06	2236	82	N	G4
18	47	M	3.91E+04	950	50	N	G2
19	65	F	1.07E+06	79.45	20	N	G3
20	63	M	5.35E+05	2320	42	N	G2
21	56	M	1.12E+05	3208	32	N	G3
22	66	F	5.22E+05	845.12	32	N	G2
23	56	F	1.82E+04	123	26	N	G2
24	64	F	1.46E+04	3445	63	N	G1

y, years; F, Female; M, Male; G1, Well differentiated; G2, Moderately differentiated; G3, Poorly differentiated; G4, Undifferentiated.

## Discussion

As an important member of the DEAD-box family, DDX17 is involved in various pathological processes, especially HCC metastasis. Xue and colleagues determined that DDX17 altered the migration and invasion abilities of HCC by altering its expression levels in HCC cell lines *in vitro* (32). Moreover, Chen and colleagues reported that DDX17 regulates the alternative splicing of PXN-AS1 to promote HCC metastasis (20). Given that chronic HBV infection is the major aetiology of HCC, we explored the functional role of DDX17 in HBV-related HCC. We demonstrated that DDX17 is positively related to HBV replication and promotes HBx-mediated HCC metastasis. Our findings are similar to those of the studies mentioned above and provide more evidence for the essential roles of DDX17 in HBV and HCC. Interestingly, in contrast to DDX17, another DEAD-box family member, DDX5, whose structure is similar to that of DDX17, is negatively related to HBV replication and inhibits HBV-related HCC development (33). Similarly, *in vitro* and *in vivo* data suggest that DDX5 dramatically inhibits HCC tumorigenesis (34). Collectively, these reports indicate that DEAD-box family members regulate HBV-related HCC in multiple ways and may have opposite effects on tumorigenesis.

As a general activator of gene expression, HBx is widely involved in the development of HBV-related HCC. HBx promotes HCC by upregulating the expression of oncogenes. A previous study showed that HBx activated the transcription of FoxM1 to promote HBx-mediated HCC metastasis, while FoxM1 inhibition significantly decreased HBx-enhanced hepatoma cell invasion *in vitro* and lung metastasis *in vivo* (35). Additionally, HBx upregulates MSL2 by activating YAP/FoxA1 signaling, and HBx-elevated MSL2 modulates HBV cccDNA in liver cancer cells, leading to hepatocarcinogenesis (36). The downregulation of tumor suppressor genes is another mechanism by which HBx promotes tumor progression. For instance, HBx was shown to reduce the level of GNA14, which promoted the proliferation and metastasis of HCC (37). In addition, HBx was shown to induce the expression of the miRNA TLRC-m0008_3p (miR-3928v) through the NF-kB/EGR1 signaling pathway, resulting in the downregulation of the tumor suppressor gene VDAC3 to accelerate the progression of HCC (38). As mentioned above, HBx participates in the development of HCC by regulating gene expression. Consistently, our results showed that HBx enhances the expression of DDX17 to promote the metastasis of HBV-related HCC. We further explored the relationship between DDX17 and HBx and found that DDX17 upregulated the expression of HBx in cells by stimulating the X promoter of HBV but did not affect the expression of exogenous flag-HBx with the CMV promoter, which differs from the X promoter. Therefore, we speculated that the X promoter is the essential site at which DDX17 regulates HBx.

ZWINT is a centromere-complex component required for the mitotic spindle checkpoint and several centromere proteins similar to ZWINT have been proven to be necessary for HCC. Centromere protein A (CENPA) is one of the most important centromere proteins in HCC. When researchers used Jumonji lysine demethylase (JmjC) inhibitors, which strongly depleted the expression of CENPA, *in vitro* and *in vivo*, HCC progression was inhibited (39). Moreover, CENPA may be a therapeutic target for HCC since its knockdown affects HCC cell proliferation (40). Another centromere protein, centromere protein M (CENPM), was shown to be overexpressed in HCC samples, and further studies found that CENPM inhibited cell apoptosis and promoted cell cycle progression by affecting P53 signaling to promote HCC. Researchers also reported that HBx decreased the expression of miR-1270 to upregulate that of CENPM and thereby promote HCC (41). Some studies have proven that the expression of ZWINT may be altered in HCC and that these changes regulate the development of HCC (42, 43). Moreover, ZWINT was determined to be the key gene involved in the molecular pathogenesis of HBV-related HCC by researchers performing an integrative analysis of all available high-throughput gene expression data on HBV-related HCC patients (44). Our study provides further evidence regarding the regulation of HBV and HCC and suggest that ZWINT can serve as a novel therapeutic target for HBV-related HCC.

Generally, our study results suggest that DDX17 plays a key role in promoting the occurrence and development of HBV-related HCC and that inhibition of DDX17 can block tumor metastasis mediated by HBx. This finding indicates that DDX17 is a potential target for the development of mechanism-based HBV-related HCC prevention strategies.

## Data Availability Statement

The datasets presented in this study can be found in online repositories. The names of the repository/repositories and accession number(s) can be found in the article/**Supplementary Material**.

## Ethics Statement

The studies involving human participants were reviewed and approved by The Clinical Research Ethics Committee of the Chongqing Medical University. The patients/participants provided their written informed consent to participate in this study. The animal study was reviewed and approved by Laboratory Animal Center of the Chongqing Medical University. Written informed consent was obtained from the individual(s) for the publication of any potentially identifiable images or data included in this article.

## Author Contributions

JC and S-TC designed the study. M-LD, XW, J-HR, H-BY, Y-PQ, ZY, M-LY, C-YZ, HZ, S-TC, and XH performed the experiments and analyses. J-HR, S-TC, and JC provided the materials. M-LD, XW, S-TC, and JC wrote the manuscript. JC and S-TC critically reviewed the manuscript. JC supervised the study. All authors contributed to the article and approved the submitted version.

## Funding

This work was supported by National Natural Science Foundation of China (81922011 and 81871656 to JC), Postdoctoral Science Foundation of China (2020M683261 to STC, BX2021400 to S-TC), Natural Science Foundation Project of Chongqing (cstc2019jscx-dxwtBX0020 to JC) and Chongqing Natural Science Foundation (cstc2021jcyj-bsh0027 to S-TC).

## Conflict of Interest

The authors declare that the research was conducted in the absence of any commercial or financial relationships that could be construed as a potential conflict of interest.

## Publisher’s Note

All claims expressed in this article are solely those of the authors and do not necessarily represent those of their affiliated organizations, or those of the publisher, the editors and the reviewers. Any product that may be evaluated in this article, or claim that may be made by its manufacturer, is not guaranteed or endorsed by the publisher.

## Abbreviations

HBV, hepatitis B virus; HBx, hepatitis B viral X protein; DDX17, DEAD-box RNA helicase 17; HCC, hepatocellular carcinoma; ZWINT, ZW10 interacting kinetochore protein; HBsAg, hepatitis B surface antigen; GAPDH, glyceraldehyde-3-phosphate dehydrogenase; NTCP, Na^+^/taurocholate cotransporting polypeptide; FBS, fetal bovine serum; AAV, adeno-associated virus; WT, wild-type; Co-IP, co-immunoprecipitation.
