# Supplementary material for: HBx Mediated Increase of DDX17 Contributes to HBV-Related Hepatocellular Carcinoma Tumorigenesis
Source: Front Immunol. 2022 Jun 16;13:871558. doi: 10.3389/fimmu.2022.871558 (PMC9243429; doi:10.3389/fimmu.2022.871558)
Supplement: Supplementary file 1 [file DataSheet_1.pdf]

Supplementary Figure 1

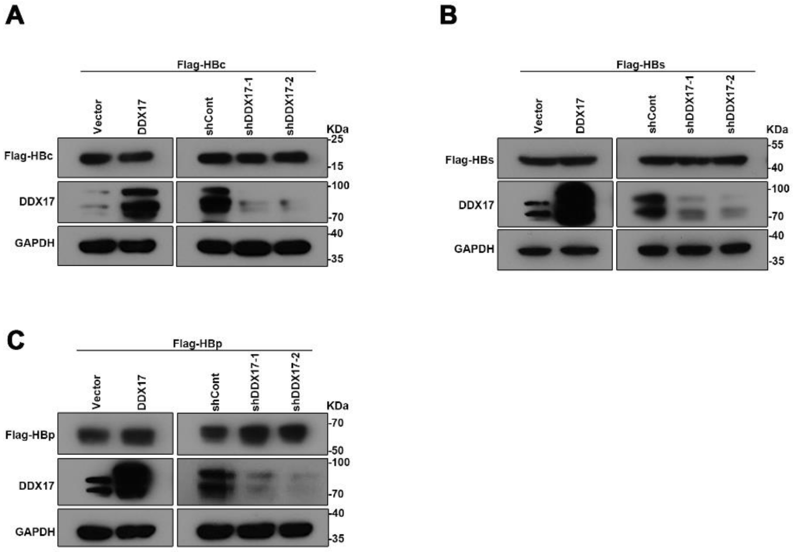

Supplementary Figure 1. The viral protein HBx is responsible for DDX17 upregulation. (A) The vector or DDX17 plasmids were co-transfected with Flag-HBc into the HepG2 cells; the short hairpin RNAs targeting DDX17 (shDDX17-1 and shDDX17-2) or nontargeting shRNA (shCont) were co-transfected with Flag-HBc into the HepG2 cells. Western blot examined the protein levels of Flag-HBc. (B) The vector or DDX17 plasmids were co-transfected with Flag-HBs into the HepG2 cells; the short hairpin RNAs targeting DDX17 (shDDX17-1 and shDDX17-2) or nontargeting shRNA (shCont) were co-transfected with Flag-HBs into the HepG2 cells. Western blot examined the protein levels of Flag-HBs. (C) The vector or DDX17 plasmids were co-transfected with Flag-HBp into the HepG2 cells; the short hairpin RNAs targeting DDX17 (shDDX17-1 and shDDX17-2) or nontargeting shRNA (shCont) were co-transfected with Flag-HBp into the HepG2 cells. Western blot examined the protein levels of Flag-HBp. GAPDH was used as the internal quantitative control.

Supplementary Figure 2

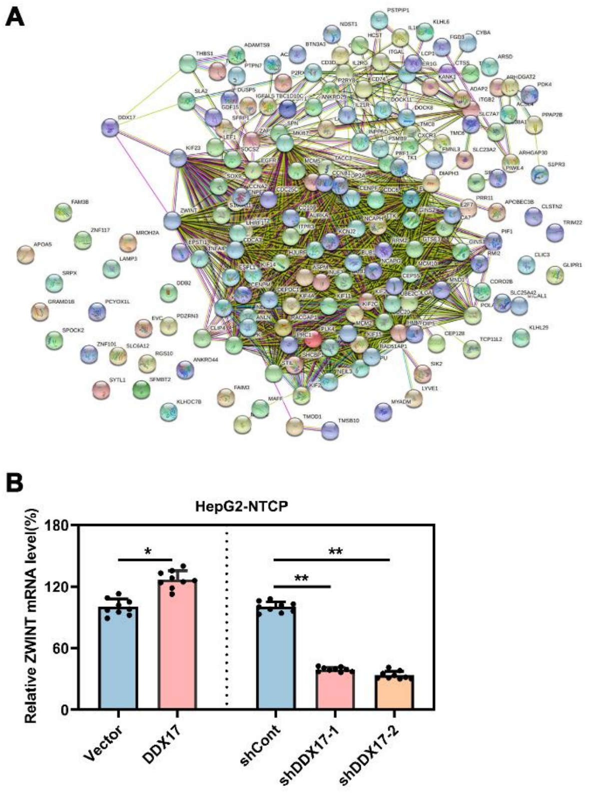

Supplementary Figure 2. DDX17 promotes HBV transcription and replication by upregulating ZWINT. (A) The protein interaction between DDX17 and 181 differential candidate genes was analyzed by string database. (B) DDX17 regulated ZWINT mRNA expression in HBV-infected HepG2-NTCP cells measured by real-time PCR assay. β-actin was used as an internal quantitative control. *P<0.05, **P< 0.01.

Supplementary Figure 3

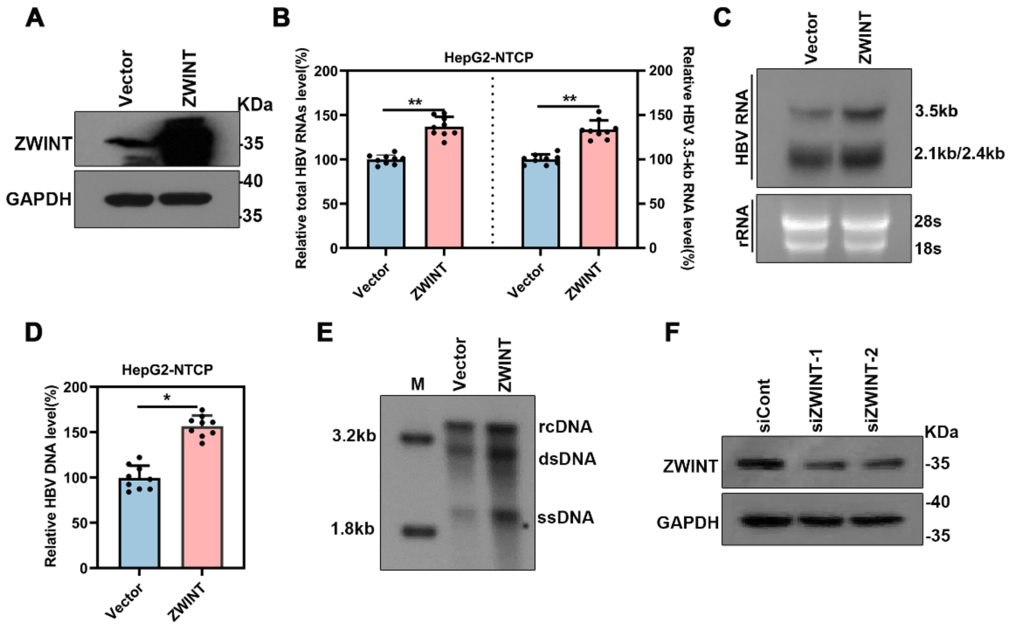

Supplementary Figure 3. ZWINT overexpression promotes HBV transcription and replication. (A) The overexpression efficiency of ZWINT was tested by western blotting analysis in HBV-infected HepG2-NTCP cells. GAPDH was used as a reference gene. (B-C) The effect of ZWINT overexpression HBV RNAs in HBV-infected HepG2-NTCP cells was analyzed by real-time PCR (B) and Northern blotting analysis (C). (D-E) The absolute quantification PCR and Southern blot were performed to determine the level of HBV core DNA after overexpression of ZWINT in HBV-infected HepG2-NTCP cells. (F) Western blotting was used to access ZWINT expression after transfected with negative control (siCont) or siRNA (siZWINT-1 and siZWINT-2) in HBV-infected HepG2-NTCP cells. GAPDH was used as the internal quantitative control. *P<0.05, **P< 0.01.

Supplementary Figure 4

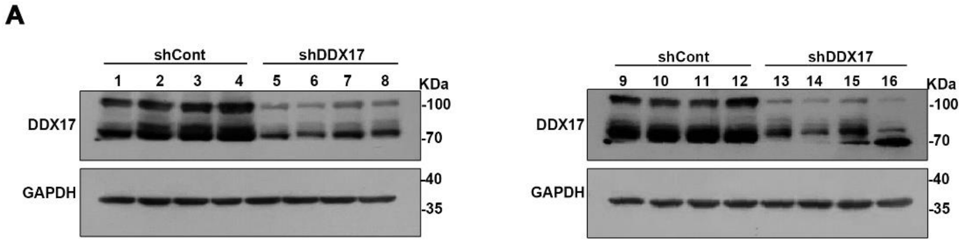

Supplementary Figure 4. DDX17 promotes HBV transcription and replication *in vivo*.(A) Western blotting was used to access DDX17 expression in liver after mice from each group treated with AAV-shCont or AAV-shDDX17. GAPDH was used as the internal quantitative control.

Supplementary Figure 5

A

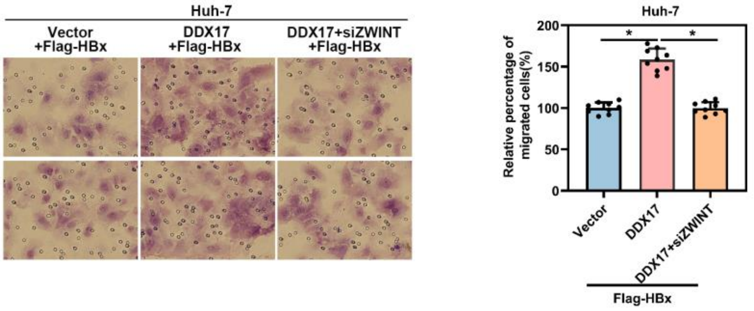

Supplementary Figure 5. Increased expression of DDX17 mediated by HBx promotes the metastasis of HBV-related HCC. (A) The effects of overexpression DDX17 meanwhile downregulation of ZWINT in Huh-7 cells for migration analyzed by transwell assays. *P<0.05.

Supplementary Table S1. The sequences of the shRNAs and siRNAs used this study

Gene	Primer Sequences (5'→3')
shDDX17-1	F：ACAAGTTGATCCAACTAATctcgagggtttttg
	R: ATTAGTTGGATCAACTTGT
shDDX17-2	F: AGAGGATTTCCTTCGTGAT
	R: ATCACGAAGGAAATCCTCT
siZWINT-1	F: CUAAUUCUCCAAGCUGAUUTT
	R: AAUCAGCUUGGAGAAUUAGTT
siZWINT-2	F: UCAUGCUAACUUUGACAGUTT
	R: ACUGUCAAAGUUAGCAUGATT

Supplementary Table S2. The sequences of the primers used this study

Gene	Primer Sequences (5'→3')
HBV 3. 5-kb RNA	F: GCCTTAGAGTCTCCTGAGCA
	R: GAGGGAGTTCTTCTTCTAGG
total HBV RNAs	F: ACCGACCTTGAGGCA TACTT
	R: GCCTACAGCCTCCTAGTACA
β-actin mRNA	F: CTCTTCCAGCCTTCCTTCCT
	R: AGCACTGTGTTGGCGTACAG
HBV DNA	F: CCTAGTAGTCAGTTA TGTCAAC
	R: TCTA TAAGCTGGAGGAGTGCGA
DDX17	F: GAACATCCGGAAGTAGCAAGG
	R: GATCCATCAACACATCCATTACATAT
ZWINT	F: CACGTAGAGGCCATCAAAATTGG
	R: CGGAGTTGTGTCCGTTTCCT
S100A4	F: AGCTAGCATGGCGTGCCCTCTGGA-3
	R: CGGATCCTCATTTCTTCCTGGGCTG
Loxl2	F: ATGAAGTCCAGTGCACAGGG
	R: CCCGGCATCTTCTTCATGGT
TGFB2	F: GCAGATCCTGAGCAAGCTG
	R: GTAGGGTCTGTAGAAAGTGG
SOx9	F: CGACTACGCTGACCATCAGA
	R: AGACTGGTTGTTCCCAGTGC
BMP2	F: GAGGTCCTGAGCGAGTTCGA
	R: ACCTGAGTGCCTGCGATACA
LEF1	F: TGTTTATCCCATCACGGGTGG
	R: TGTTTATCCCATCACGGGTGG
TGFB1	F: TGGTGGAAACCCACAACGAA
	R: GAGCAACACGGGTTCAGGA
TGFBR1	F: TCAGGTTCTGGCTCAGGTTT
	R: TTCTCCAAATCGACCTTTGC

WNT4	F: AGACGTGCGAGAAACTCAAAG
	R: GGAACTGGTATTGGCACTCCT
HIF1A	F: AGCTTCTGTTATGAGGCTCACC
	R: TGACTTGATGTTCATCGTCCTC
AKNA	F: CGACGCGTGCGTGGAGGTATAGGTGTGCAG
	R: CCGCTCGAGATGGGACCTGGCCTGGCTT
ENG	F: ACCACAGCGGAAAAAGGTGC
	R: GGTAGAGGCCCAGCTGGAA
