# Supplementary material for: HBx Mediated Increase of DDX17 Contributes to HBV-Related Hepatocellular Carcinoma Tumorigenesis
Source: Front Immunol. 2022 Jun 16;13:871558. doi: 10.3389/fimmu.2022.871558 (PMC9243429; doi:10.3389/fimmu.2022.871558)

Original figures of blots 1

Figure 2A

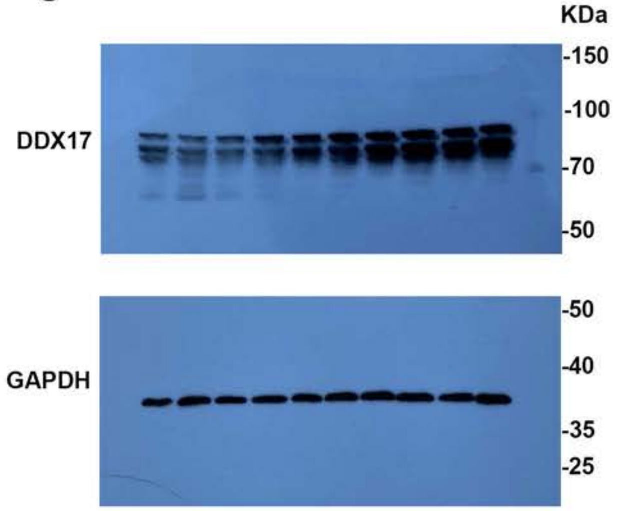

Figure 2C

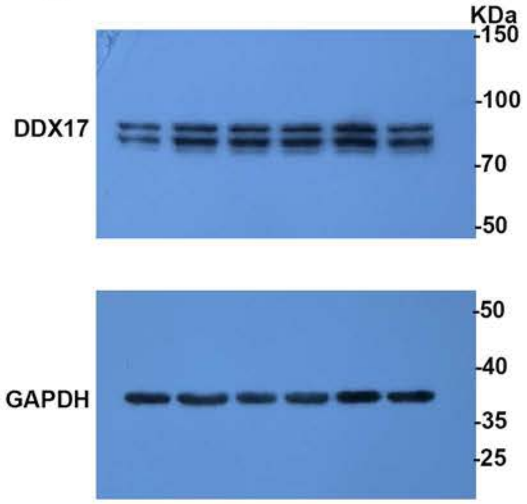

Figure 3B

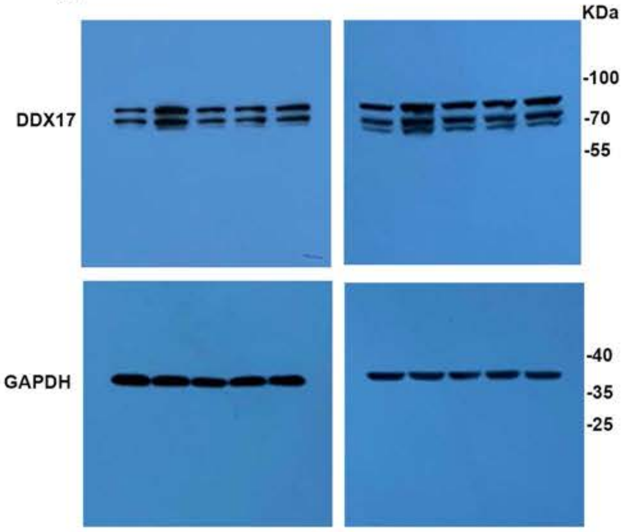

Figure 3D

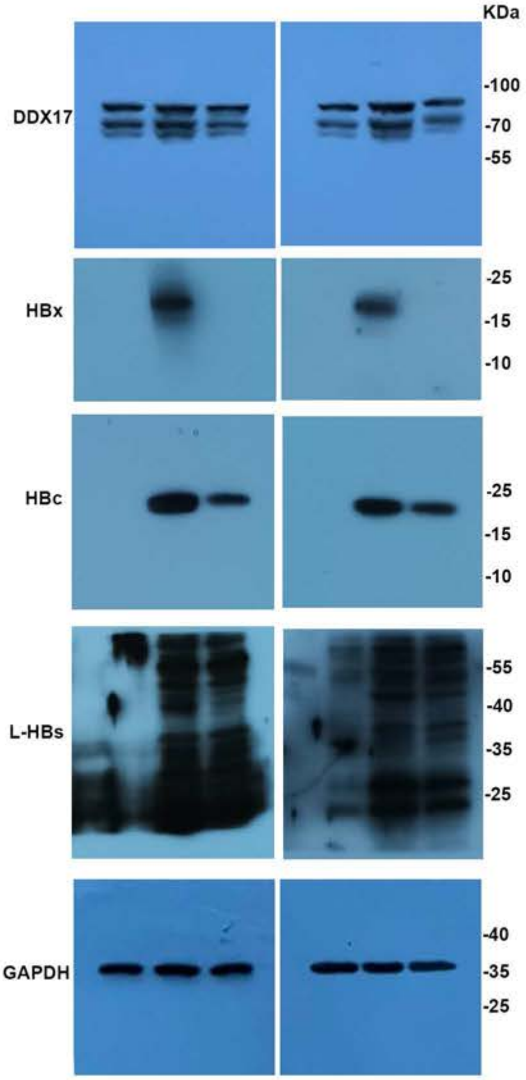

Figure 3F

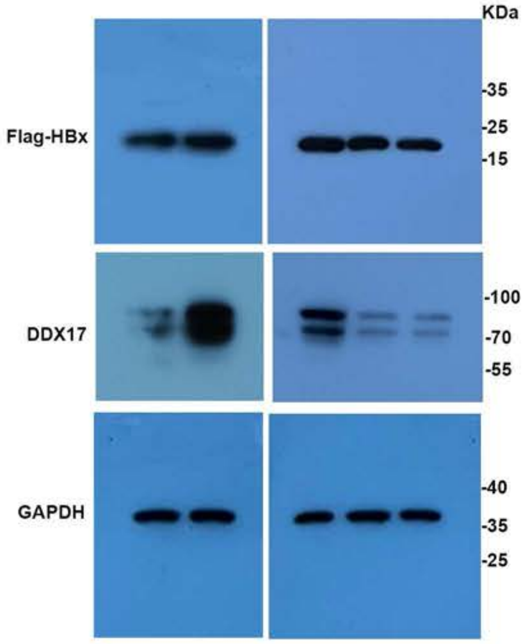

Original figures of blots 2

Figure 4A

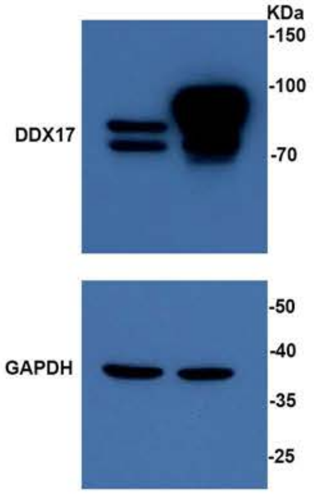

Figure 4C

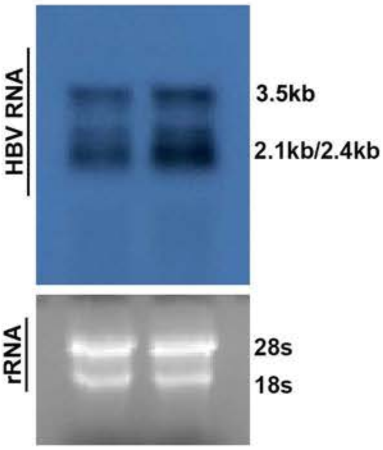

Original figures of blots 3

Figure 4E

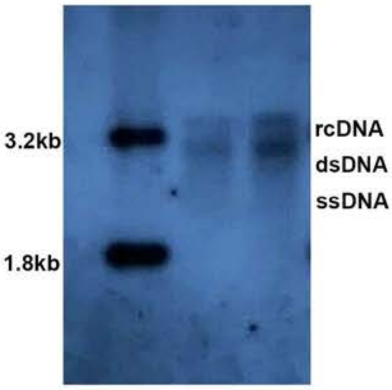

Figure 4F

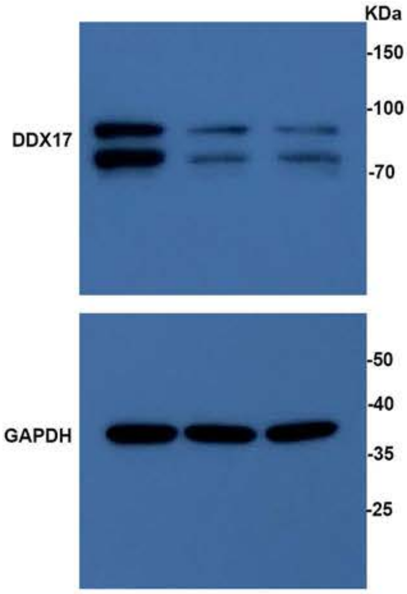

Figure 4H

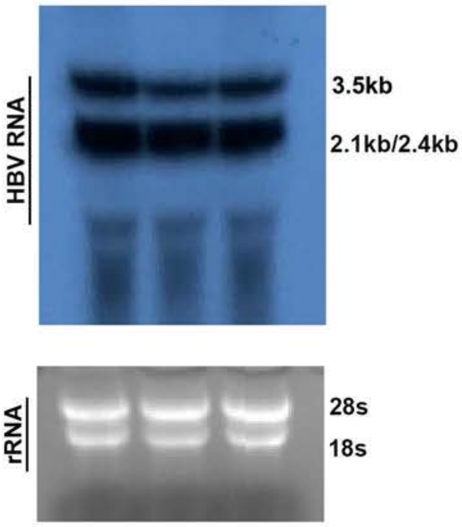

Figure 5C

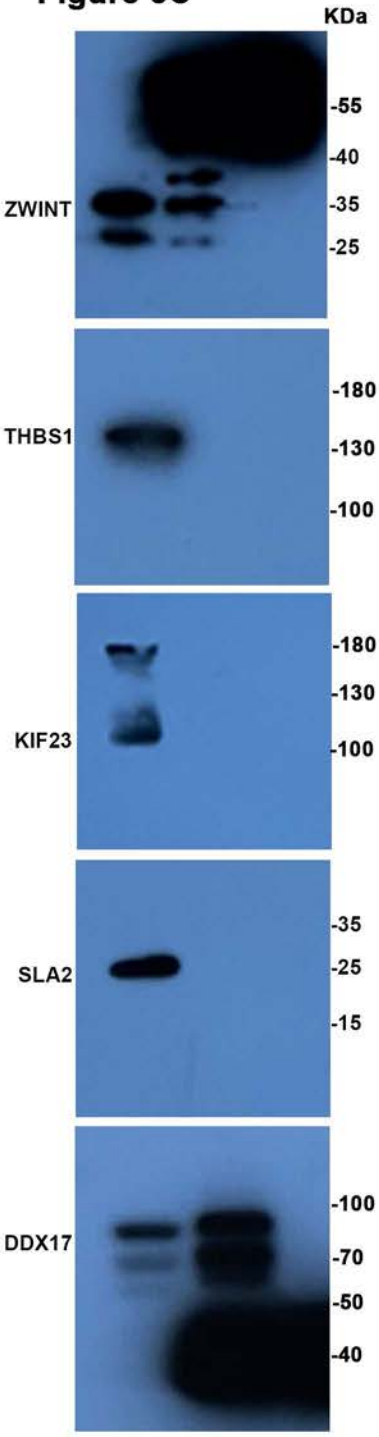

Figure 4J

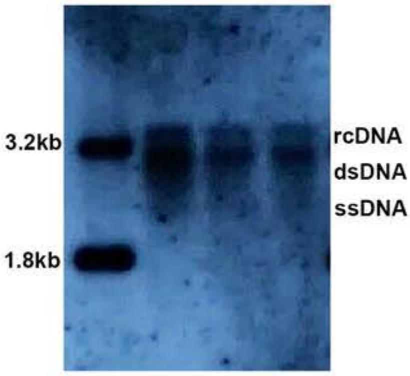

Figure 5D

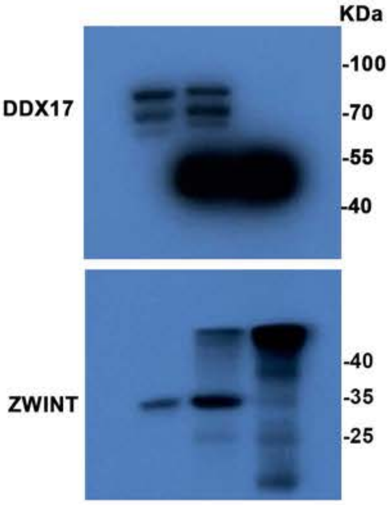

Figure 5E

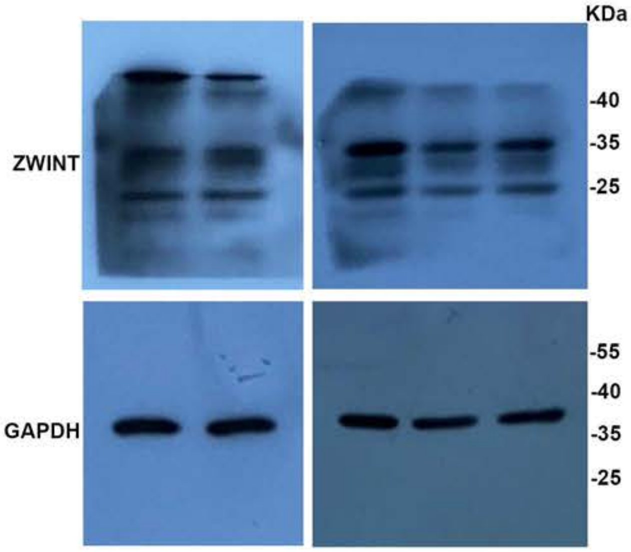

Original figures of blots 4

Figure 5G

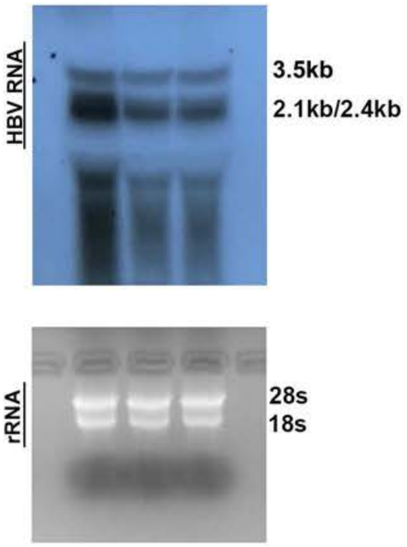

Figure 5I

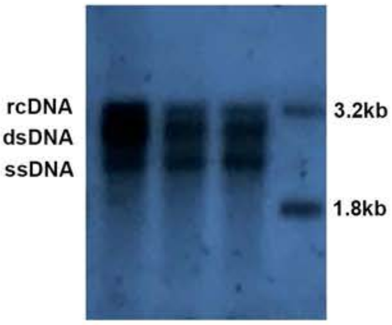

Figure 6F

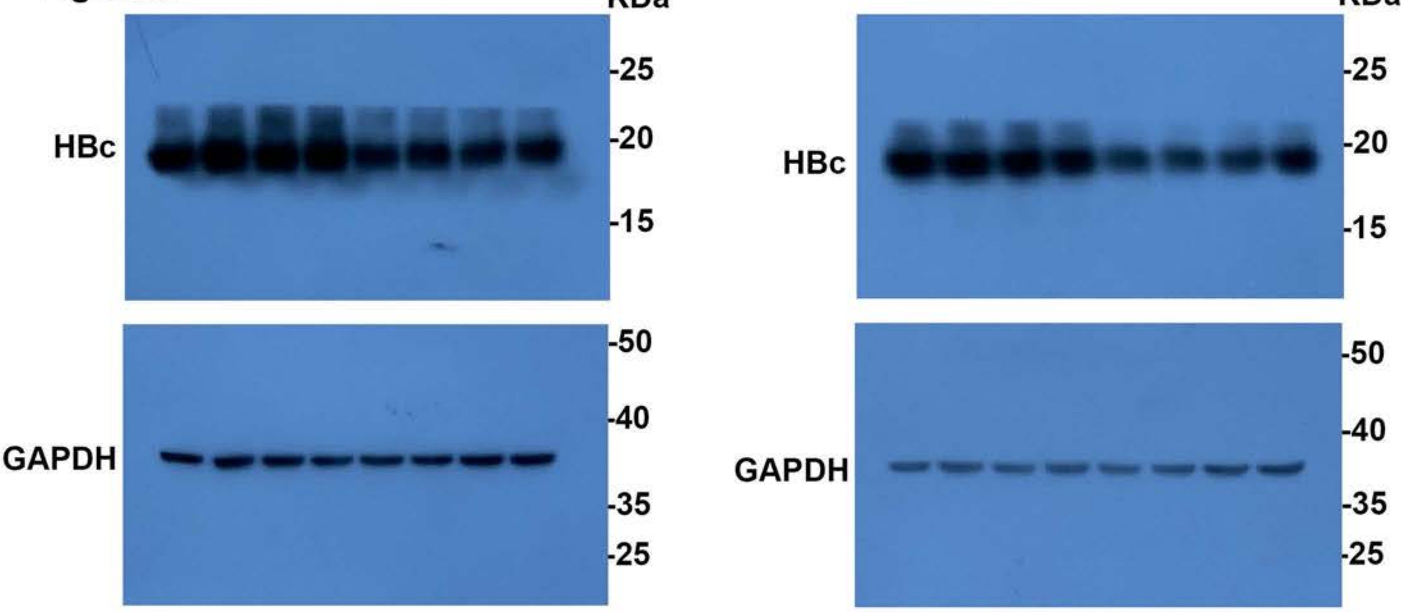

Figure 8A

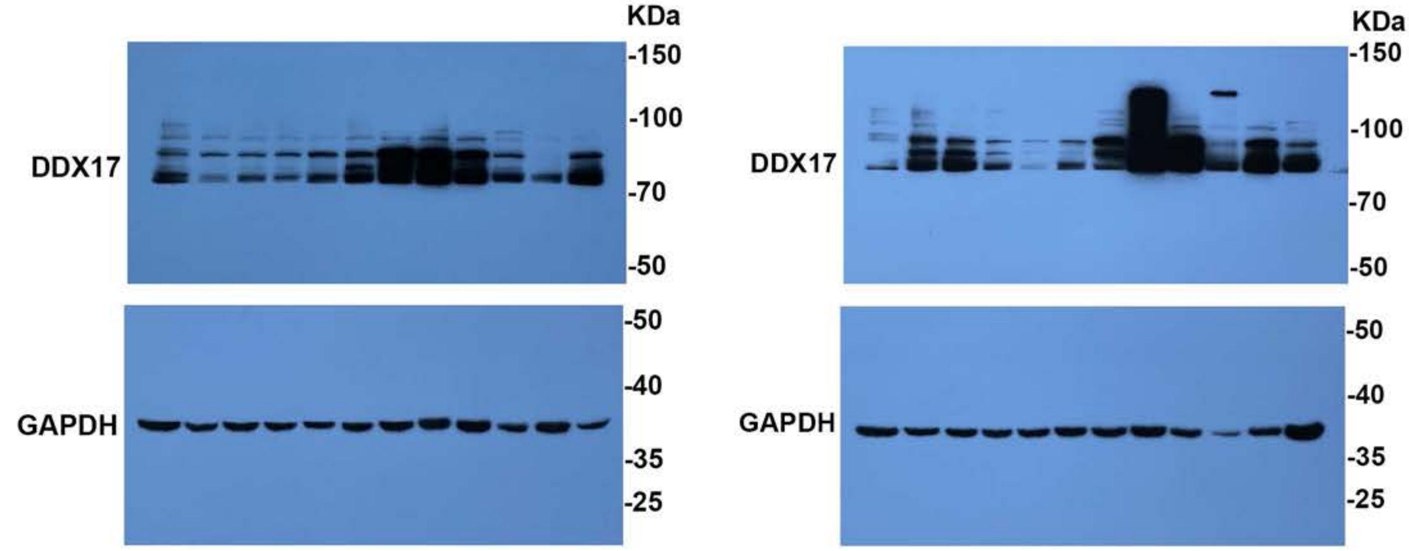

Original figures of blots 5

Sipplementary FIgure 1A

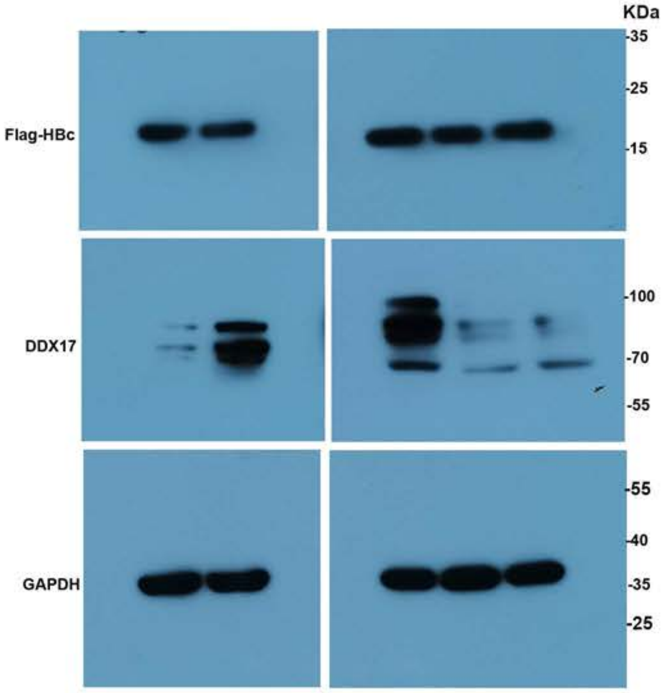

Sipplementary FIgure 1B

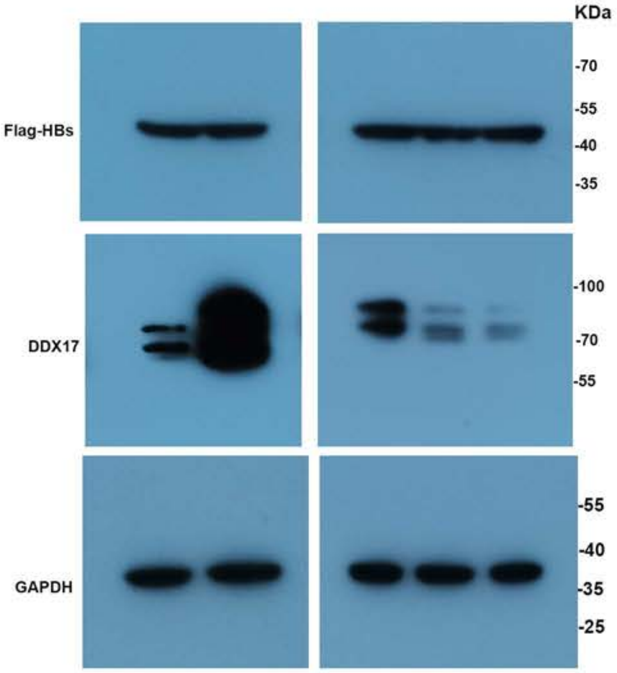

Sipplementary FIgure 1C

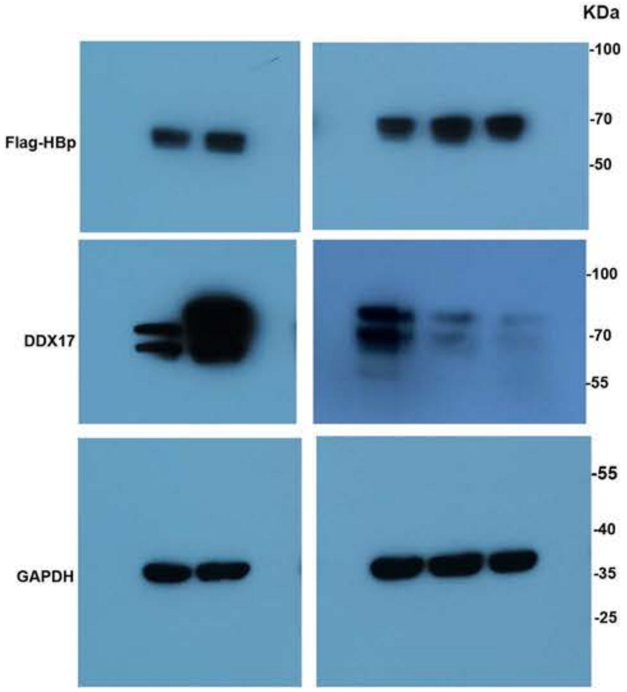

Original figures of blots 6

Supplementary Figure 3A

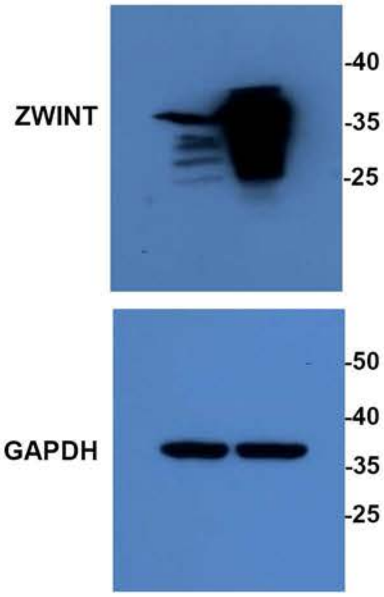

Supplementary Figure 3C

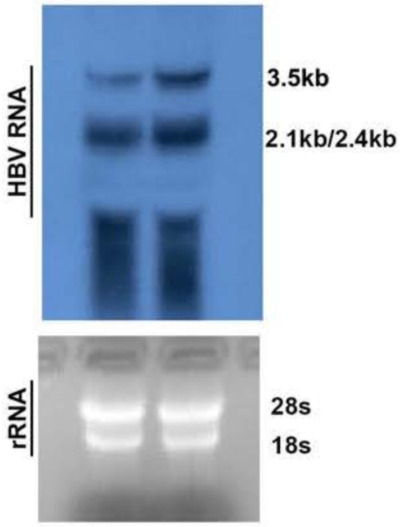

Supplementary Figure 3E

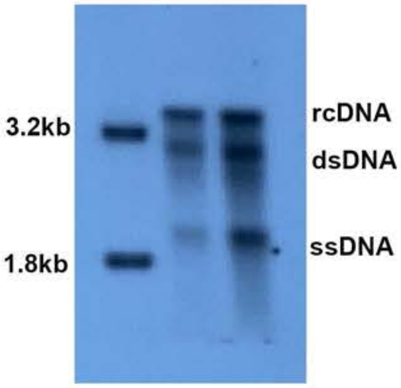

Supplementary Figure 3F

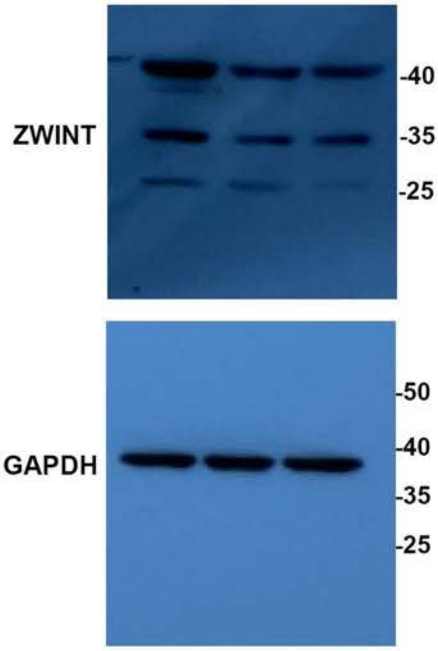

Original figures of blots 7

Supplementary Figure 4A

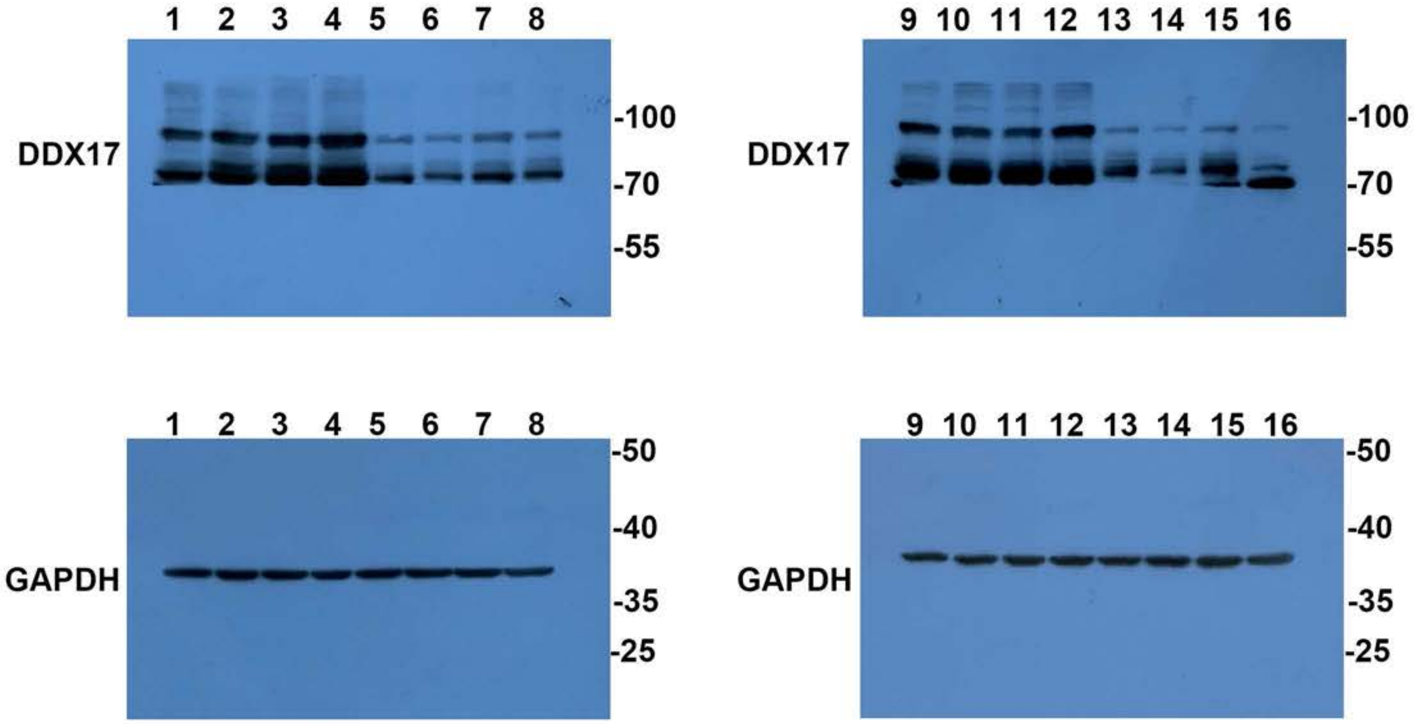

Supplement: Supplementary file 3 [file DataSheet_3.pdf]
